# Early Exotic Vegetation Development Is Affected by Vine Plants and Bird Activity at Rapidly Exposed Floodplains in South Korea

**DOI:** 10.3390/biology12050696

**Published:** 2023-05-09

**Authors:** Jae-Hoon Park, Ji-Won Park, Yoon-Seo Kim, Jung-Min Lee, Eui-Joo Kim, Bo-Yeon Jeon, Se-Hee Kim, Young-Han You

**Affiliations:** Department of Life Science, Kongju National University, Gongju 32588, Republic of Korea; kn5314@smail.kongju.ac.kr (J.-H.P.); ecopark@kongju.ac.kr (J.-W.P.); 201502761@smail.kongju.ac.kr (Y.-S.K.); ljm@smail.kongju.ac.kr (J.-M.L.); euijoo@kongju.ac.kr (E.-J.K.); jbytit17@smail.kongju.ac.kr (B.-Y.J.); ksh41631@smail.kongju.ac.kr (S.-H.K.)

**Keywords:** aquatic ecosystem, avifauna, flora, sand bar, wetland

## Abstract

**Simple Summary:**

In a river system where material flow is dynamic, the expansion of exotic plants through seed dispersion is affected by physical and biological factors, such as water, wind, and animal activities. In this study, the relationship between the life forms of plants and terrestrial birds that carry plant seeds in the establishment of exotic plants in exposed areas that were originally submerged and exposed to a drop in water level was analyzed. As a result, the development of exotic plants was promoted in proportion to the area of vine plants and the population of small terrestrial birds. This provides insight not only about the life forms of existing plants, but also the landscape of potential bird habitats, including vegetation patches; these should be considered for the effective management of exotic plants along rivers.

**Abstract:**

For the study on the relationships between the seed dispersal of exotic plants and bird population, flora, avifauna, vegetation patches, and the dynamics of seed banks were investigated in and around the exposed floodplains of the large rivers, and the causes of exotic vegetation development were determined with respect to plant life form, bird population characteristics, and landscape using multivariate analysis. The number of dominant exotic plant species observed in exposed areas was higher than that observed in an abandoned field and paddy field undergoing secondary succession. Additionally, the area occupied by exotic vegetation in exposed areas increased with the increase in number of vine plants and small terrestrial birds, whereas the relationship between vine and runner plants was inversely proportional. Therefore, to control exotic plants in exposed floodplains surrounding large rivers, it is necessary to remove vines and shrubs along the waterfront where small resident birds carrying plant seeds live and to maintain and manage runner plant populations. Furthermore, implementing an ecological landscape management strategy, such as afforestation through the planting of trees, may also be effective.

## 1. Introduction

In riparian ecosystems, floodplains are formed by the deposition of sediments and provide a habitat for various organisms [1]. The ecological functions of riparian and terrestrial ecosystems are interlinked due to their geographical adjacency to a floodplain [2]. Owing to the spatiotemporal dynamics of riparian ecosystems, the interactions between the ecotones formed within the floodplains become structural and functional elements of these areas [3]. Sedimentation in floodplain maintains the high lotic, lentic, and semi-aquatic habitat diversity through river dynamics [3].

Rivers serve as crucial pathways for the distribution and migration of aquatic plants [4], thus affecting the succession of this vegetation. However, rivers can also inhibit seed germination and development due to physical factors, such as rapid flow and droughts, particularly during the emergence of seedlings [5,6]. Wetland plant seeds have adapted to reach and accumulate in areas optimal for germination and growth [7], making seed dispersal important for their gene flow [8]. Generally, seeds dispersed by animals have stronger genetic connectivity than those by water or wind [8]. However, their success rate can be affected by factors such as the dispersal ability of each species and the regional stability for survival after dispersion [9].

Currently, as urbanization advances, riparian ecosystems are increasingly impacted by anthropogenic disturbances, such as vegetation destruction, introduction of exotic species, waterway opening, cultivation, and road construction, owing to continuous development [10]. These disturbances have negatively affected water resource utilization and environmental functions by disrupting river channels, drawing public attention to the pressing need for river restoration [10].

To address this issue, the South Korean government promoted large-scale river restoration projects, including the construction of the Gongju, Sejong, and Seungchon weirs, with the aim of securing water sources, providing flood control, improving water quality, developing spaces for cultural and leisure activities, and supporting local development around large rivers [11]. However, researchers have not reached a consensus on the positive and negative impacts of these projects on riparian ecosystems [10,12]. Several years after their construction, the weirs were opened to allow water flow for the purpose of improving water quality. This action, however, led to an unintended consequence: an increase in the inflow of exotic plants and pollution in exposed areas due to high concentrations of accumulated organic matter. As a result, it has become essential to identify and manage the causes of exotic plant introduction.

Thousands of plant species worldwide have adapted to freshwater wetlands, resulting in diverse and highly productive ecosystems [13]. However, the increased frequency and duration of flooding events due to changes in land use patterns, climate change-induced extreme weather, and regime alterations stemming from reservoir and weir development within a floodplain can disrupt the soil environment by introducing contaminants [14]. Physical disturbances in an ecosystem also affect the succession of vegetation [15]. Previous studies have demonstrated that such extreme drought and flooding related soil disturbances affect succession through vegetation growth and survival [15].

An invasive plant is an organism that spreads through the formation of an exotic population, often introduced by human activities [16]. These plants can cause massive, rapid, and irreversible changes in the native communities [16]. Due to the global threat posed by the invasive plants to the native ecosystem, many studies have recently been conducted on their impact, distribution, and management [17,18,19,20,21,22].

Plant dispersal can also be affected by the growth of surrounding vegetation. *Sicios angulatus* L., a widely known representative exotic species in the temperate seasonal forests of South Korea and Japan, uses vines to cover river areas and form populations. Dominance of *S. angulatus* in the Tama River, with the exception of *Miscanthus sacchariflorus* (Maxim.) Hack, severely impacted the development of numerous native plants that had previously grown naturally in the understory vegetation [23]. Consequently, disturbances caused by the potential invasion of exotic species competing with the native species threaten the unique ecosystem.

Vegetation studies in South Korea indicate that the third largest number of exotic plant species have been introduced in the lotic zone, while a relatively smaller number has been introduced in the lentic habitats [20]. Therefore, the formation of early pioneer lotic communities is expected to be severely affected by the introduction of exotic plant species. This study aimed to analyze the effects of vegetation development and bird activity on the inflow of exotic plants. Additionally, the study sought to identify the cause of this inflow and propose a floodplain management plan for areas impacted by the opening of the weirs.

## 2. Materials and Methods

### 2.1. Study Area

**Figure 1:**
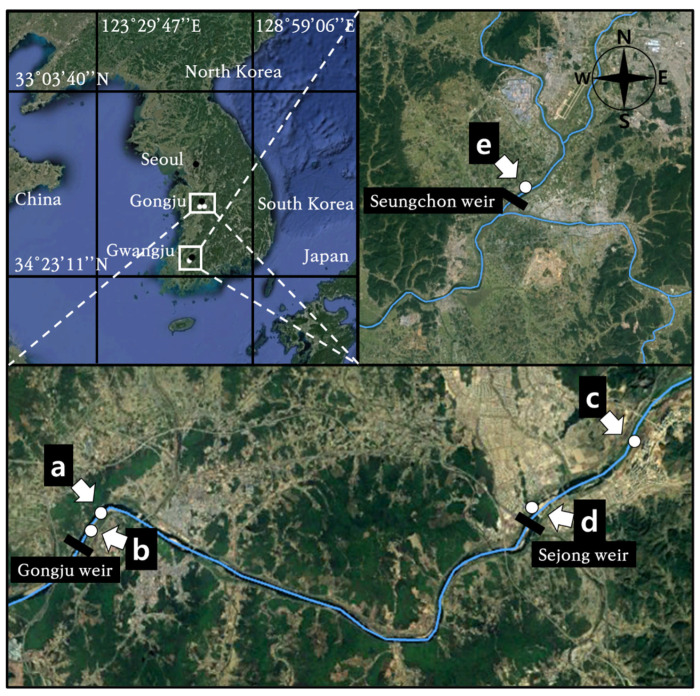
The key map of study area showing upper (**a**) and lower sites (**b**) of the Gongju weir, upper (**c**) and lower sites (**d**) of the Sejong weir, and the researched site of Seungchon weir (**e**). Blue lines on the map represent the river lines.

This study was conducted in the floodplains of Gongju, Sejong, and Seungchon weirs, located in the northern hemisphere, in the temperate seasonal forests of South Korean rivers (Figure 1). Three weirs were constructed in 2012 impound water. For the first time since their construction, floodplain sediments from the three weirs were exposed to the atmosphere after the drawdown of the weirs in the spring of 2018.

**Figure 2:**
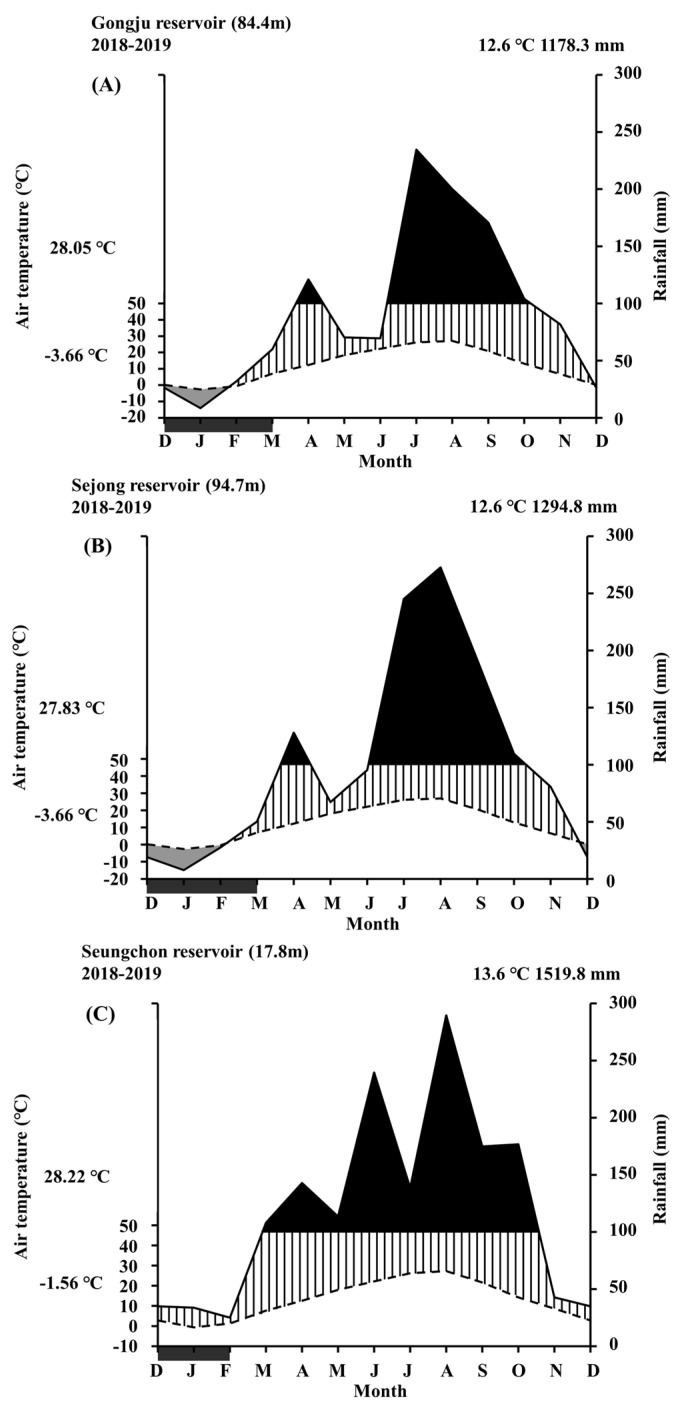
Climate diagrams for Gongju (**A**), Sejong (**B**), and Seungchon (**C**) weirs during 2018–2019. The spotted and bold lines represent the monthly average air temperature (°C) and rainfall (mm), respectively. The black colored and lined areas represent the period of rainfall (mm) over and under 100 mm, respectively, and the gray colored area represents a dry period. The darkish area within the *x*-axis shows the period during which the average daily air temperature (°C) was under 0 °C. Upper and below temperatures on the *y*-axis are the monthly maximum and minimum temperatures.

Temperature and precipitation data for the three weirs were analyzed using daily average values obtained from the ‘Open MET Data Portal’ website of the Korea Meteorological Administration [24]. Daily precipitation, measured between June and September, peaked at an average temperature of approximately 23 °C; heavy rainfalls exceeding 100 mm were recorded at temperatures ranging from 18 to 25.6 °C (Figure 2).

At the beginning of the exposed area formation, bird activity is considered to directly influence the early stages of succession that occurs in bare areas devoid of vegetation by transporting plant seeds. The plants that appear at this time are pioneers that first flowed into these areas. Seed banks, formed after vegetation has stably developed, are considered to be influenced by bird activity. Based on this concept, research on avifauna, flora, vegetation, and seed banks was sequentially conducted.

### 2.2. Vegetation Study

**Figure 3:**
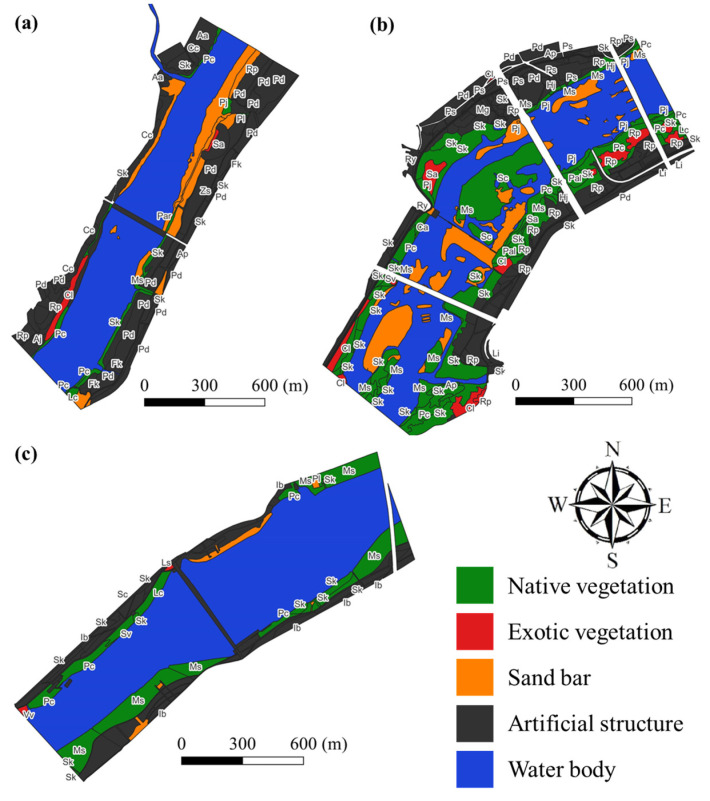
Vegetation and landscape maps for Gongju (**a**), Sejong (**b**), and Seungchon (**c**) weirs during the autumn season.

Research of the dominant vegetation in the floodplains exposed due to the drawdown of the Gongju, Sejong, and Seungchon weirs was conducted in September 2020. To this end, a map was sketched of the landscape first in the target area for the 1 km upstream and downstream sections, and then field research was conducted to create a revised landscape map using the QGIS program (version 3.26.1-Buenos Aires). Additionally, the landscape properties were categorized as native vegetation, exotic vegetation, sandbar, artificial structure, and water body (Figure 3). To identify the plant species present in the soil seed bank, a surface layer of 0–10 cm was collected from spatially low and high levels of each floodplain, and germinated species were recorded in the spring of 2021.

The flora in the three weirs were investigated for the exotic plants in their floodplains between April 2018 and November 2019. The plant species were identified and analyzed using the line transect method, where all species appearing along the line drawn across the river were recorded. Additionally, onsite monthly monitoring research was conducted to observe the species that appeared over time. The studied line transects were set up at five sites in the Gongju weir, seven in the Sejong weir, and four in the Seungchon weir.

Classification of exotic species, prepared according to the exotic species list in the ‘Nature’ website of Korea National Arboretum [25], was used to organize the data. Following Lee’s method [26], the plant life forms were largely classified based on seed dispersal, growth forms, and rhizome development, including Raunkiær’s life form [26]. This study used wind-, water-, animal-, mechanically, and gravitationally dispersed plants, vines, runners (with stolon), rosettes, and rhizomatous plants. Scientific names of the plant species followed the ‘National list of species of Korea’ produced by the government of South Korea [27].

### 2.3. Avifauna

Bird monitoring was conducted once a month from July–September 2018 using two survey methods. A full survey was conducted on the waterfront of the intensive survey area. In the outer area, the line census method was used to record all bird species and the number of individual birds seen or heard in both directions around the target site while moving along the riverside [28]. The point count method was used in two to three selected areas centered around the confluence of the rivers. In this method, all bird species and populations in the survey area were recorded by visual observation or identification of bird songs during a 15 min period [28]. Bird species observed in the research areas were identified and then organized based on Korean name, status, and body size, according to the methods described in a study [29]. Scientific names of the bird species followed the ‘National list of species of Korea’ produced by the government of South Korea [27]. Ecologically, bird body size can impact seed dispersal, as it is related to seed dispersal distance and bird habitat [30,31]. However, it is not clear which size is ecologically, quantitatively meaningful. Thus, in this study, considering that the body size of the observed birds is divided into two discontinuous groups around 40 cm (Table S3), birds having a body size less than 40 cm were classified as small birds, whereas those having a body size more than 40 cm were classified as large birds.

### 2.4. Statistical Analysis

To understand how the inflow of exotic plants is affected by the vegetation development and bird diversity, Discriminant Analysis for Principal Component (DAPC) was performed using the Statistica software program (version 7) on the vegetation data obtained from the floodplains. Pathway analysis was conducted to examine the impact of seed dispersal types and the external morphology of vegetation on the development of exotic plant species. This analysis was carried out using the JASP statistical software (version 0.14.1). The input data for the pathway analysis consisted of the number of species dispersed by water/wind, animals, mechanical trait, and gravity, as well as vine, runner, rosette, and rhizomatous plants. Additionally, the ratio of exotic to native plant species was also considered. The collected data was organized sequentially as per observational time.

Vegetation development is known to be affected by bird migration patterns in the area and seasonal changes, especially given that the plants in the temperate seasonal forests of the northern hemisphere die due to seasonal changes post September. As such, data after September was excluded from the DAPC analysis. All the data was standardized before conducting path and DAPC analysis to eliminate errors generated by variations in units of each variable [32].

## 3. Results

**Figure 4:**
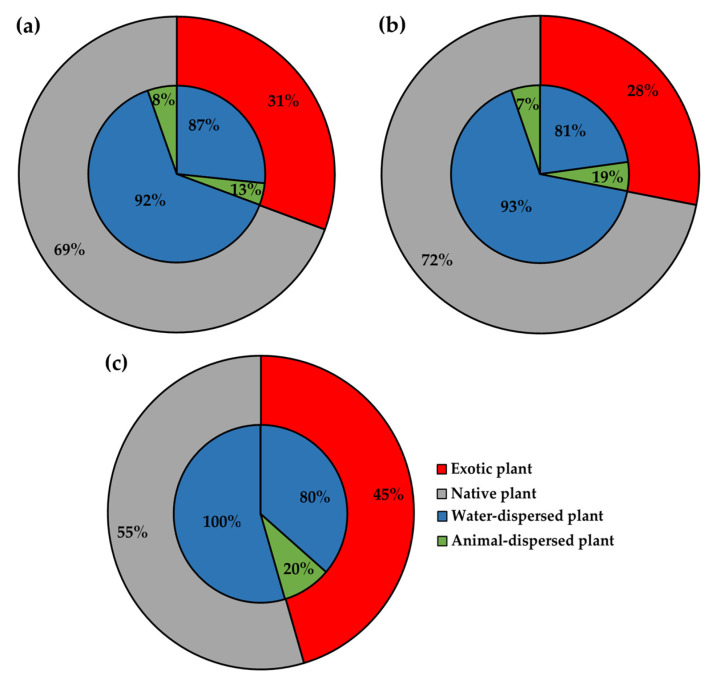
Ratio (%) of appeared number of exotic and native species at each researched area within the floodplains of Gongju (**a**), Sejong (**b**), and Seungchon (**c**) weirs.

As a result of the vegetation research, exotic plant species were more dominant in the exposed floodplains surrounding the large rivers in this study than that previously observed in the early stages of terrestrial secondary succession [33,34]. The exposed areas around the Gongju and Seungchon weirs were each occupied by two exotic plant species, while four species were found in the exposed area around the Sejong weir. The ratio of exotic plant species was 28% around the Gongju and Sejong weirs and 45% around the Seungchon weir (Figure 3 and Figure 4). Seven dominate native species were found around the Gongju and Seungchon weirs, while twelve native species were found around the Sejong weir. The ratio of appeared native plant species around the Seungchon weir was 55% and that around the Gongju and Sejong weirs was 72% (Figure 3 and Figure 4).

**Figure 5:**
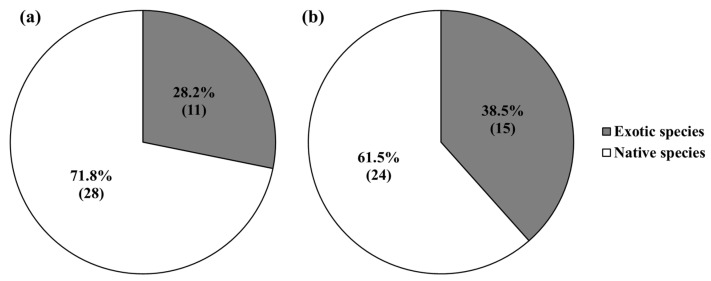
Ratio of the appeared number of exotic and native plant species within the collected seed banks at the spatially low (**a**) and high (**b**) levels of the floodplains. The numbers in the brackets are the no. of appeared plant species.

In exposed areas, exotic plants were dispersed by animals, as well as water, while native plants were almost dispersed by water alone (Figure 4). Among the ratio of appeared exotic plant species, the portion distributed by water was 80% around the Seungchon weir and 87% around the Gongju weir, while the ratio of plant species distributed by animals was 13% around the Gongju weir and 20% around the Seungchon weir (Figure 4). Among the ratio of appeared native plant species, the portion distributed by water was 92% around the Gongju weir and 100% around the Seungchon weir, while the vegetation area distributed by animals was 0% around the Seungchon weir and 8% around the Gongju weir (Figure 4). The ratios of exotic and native plant species within the seed bank were 28.2% and 71.8% in the low level floodplains and 38.5% and 61.5% in the high level floodplains (Figure 5).

The study, conducted in the lotic area of the rivers, observed a higher number of terrestrial birds than waterbirds among those classified as small birds in the exposed floodplains (Table S3). In contrast, the number of waterbirds was higher than that of the terrestrial birds among those classified as large birds (Table S3). The bird recording result shows that there were 1905 individual birds in exposed areas of the floodplains, of which 1104 were classified as small birds (Table S3). Of the small birds, 1003 were terrestrial birds and 101 were waterbirds (Table S3). Among the remaining 801 large birds, 259 terrestrial birds and 542 waterbirds were observed (Table S3).

**Figure 6:**
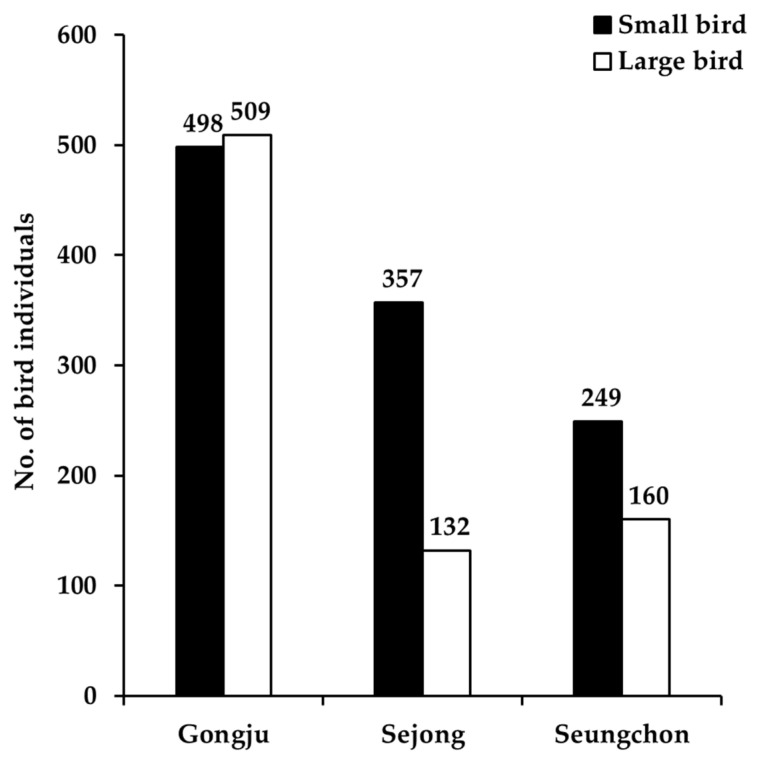
The number of observed small and large individual birds around Gongju, Sejong, and Seungchon weirs. Small and large birds mean their body size is 0–40 cm and >40 cm, respectively.

The number of birds also varied according to the research site. The total number of birds observed around the Gongju weir was 1007, comprising 498 small and 509 large birds (Figure 6). The total number observed around the Sejong weir was 489 birds, with 357 small and 132 large birds (Figure 6). The total number of birds observed around the Seungchon weir was 409, of which 249 were small and 160 were large (Figure 6).

**Figure 7:**
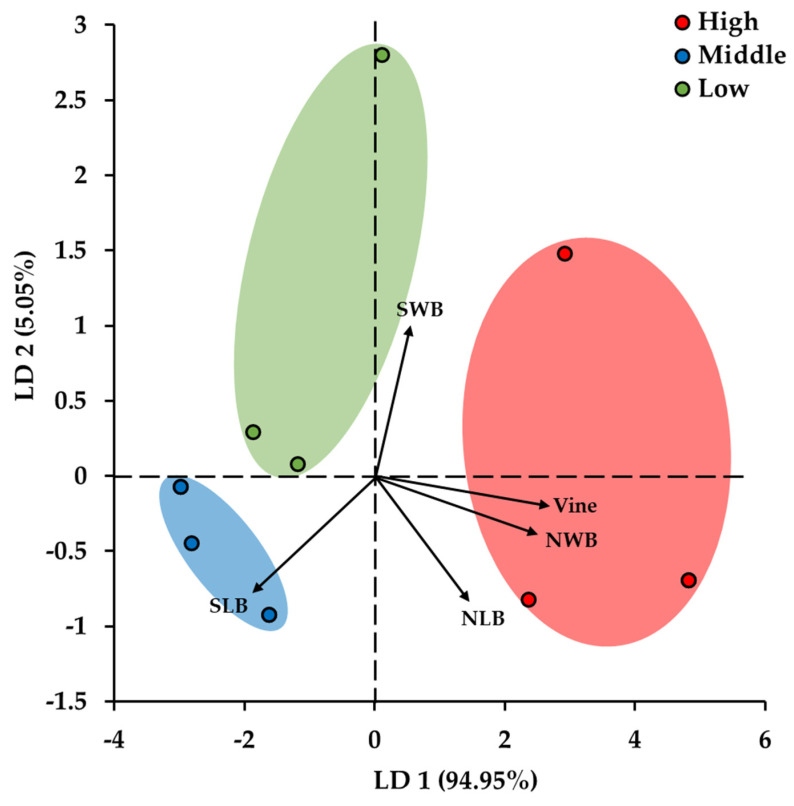
The result of DAPC analysis between the ratio of high, middle, and low exotic vegetation areas per total researched area and observed terrestrial bird individual number (NLB) and body size (SLB), water bird individual number (NWB), and body size (SWB) and ratio of dominant vine vegetation areas (Vine).

The study found that the number of exotic plant species and the area of exotic vegetation both increased in proportion to the number of small birds (Figure 7). The DAPC results revealed clear divisions among the high, middle, and low sites of exotic vegetation for the entire study area (Figure 7). Distribution was affected by the number and body size of terrestrial birds and waterbirds (Table 1).

The distribution area of vine plants increased with the increase of terrestrial birds and waterbirds (Figure 7). However, it decreased with the increase in the body size of terrestrial birds (Table 1). Furthermore, there was no correlation with the body size of waterbirds, as demonstrated by the right-angled relationship on the graph (Figure 7).

**Figure 8:**
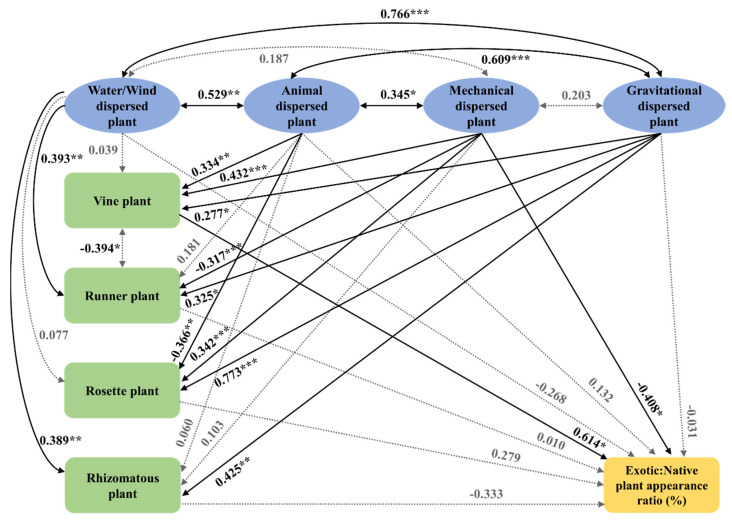
Path analysis results for the exotic native plant appearance ratios (%) based on the variety of types of seed dispersals (water- or wind-, animal-, mechanically, and gravitationally dispersed plants) and the plant growth types (vines, runners, rosettes, and rhizomatous plants). The double arrows mean correlation and the single directed arrows mean regression. The dotted lines mean the relationships have no significance. The numbers on the arrows mean standardized coefficients (β) and the *, **, and *** mean the significance probability *p* < 0.05, *p* < 0.01, and *p* < 0.001, respectively.

The activities of large animals, such as birds transporting seeds, along with vegetation dynamics within the exposed floodplain around a large river, can promote the inflow of vine plants. Consequently, exotic plants become established within the floodplains (Figure 8); however, runner plants can compete with vine plants and inhibit their development (Figure 8). Except for the vines displaying a negative correlation with the runners (−0.394 of β, *p* ≤ 0.05) in path analyses (Figure 8), no correlation was discerned between vines, runners, rosettes, and rhizomatous plants (Figure 8). The number of runners (0.393 of β, *p* ≤ 0.01) and rhizomatous plants (0.389 of β, *p* ≤ 0.01) was seen to increase with that of the wind- or water-dispersed plants (Figure 8). However, the number of vines increased (0.334 of β, *p* ≤ 0.01) and that of rosettes decreased (−0.366 of β, *p* ≤ 0.01) with an increase in the animal-dispersed plants (Figure 8). Similarly, a greater number of vines (0.432 of β, *p* < 0.001) and rosettes (0.342 of β, *p* < 0.001), and a smaller number of the runners (−0.317 of β, *p* < 0.001), were reported for a higher number of mechanically dispersed plants (Figure 8). Such correlation was seen between the number of vines, runners, rosettes, and rhizomatous plants and the gravitationally dispersed plants with the number of vine (0.277 of β, *p* < 0.05), runner (0.325 of β, *p* < 0.05), rosette (0.773 of β, *p* < 0.001), and rhizomatous plants (0.425 of β, *p* < 0.01), corresponding to the increase in the gravitationally dispersed plants (Figure 8). It can also be seen from Figure 8 that the appearance ratio (%) of exotic plants increased (0.614 of β, *p* < 0.05) with an increase of vines but decreased (−0.408 of β, *p* < 0.05) with an increase of mechanically dispersed plants (Figure 8).

## 4. Discussion

Vegetation research in the exposed floodplains surrounding the large rivers in the study area revealed a total of six dominant species, with two to four exotic species present in each location in the second year after exposure (Table S1). Furthermore, the ratio of appeared plant species was as high as 45% in one region (Figure 3 and Figure 4). These results were almost consistent with the ratio of exotic plants in the seed banks (Figure 5 and Table S2). Findings in this study differ from those of previous studies on paddy fields that reported either no domination or domination by only one plant species [33,35,36], and in case of an abandoned field, the ratio of appeared exotic plant species that was undergoing secondary succession in a temperate seasonal forest, similar to that of this study area, was only 15% [37]. In comparison to the previous studies, our results revealed an increased number of dominant exotic plant species and ratio of appeared exotic plant species in the exposed floodplains within the study area.

Analysis of plant dispersal mechanisms revealed a correlation between the method of dispersal and the type of plant found in the exposed floodplains (Figure 8). These results indicate that the plant dispersal means differed from those previously observed. The form of dispersal is typically determined by seed morphology and has adapted over time in response to physical or biological vectors; however, recent studies have shown that plants can adjust their dispersion in response to different environmental conditions [38] and are capable of altering their shape and that of their seeds to change the dispersal method [39].

The results of this study showed that the number of gravity-dispersed plant species increased in proportion to the number of wind- or water-dispersed plant species (Figure 8). This trend is probably related to the habitat characteristics in the study area. The land upstream from the weirs was flooded for six years prior to the drawdown, and the opening of the weirs resulted in the rapid exposure of the floodplains as the water level decreased. Subsequently, the process of primary succession began due to the absence of seeds in the newly exposed soil. Therefore, the gravity-dispersed plant species identified in the study area were introduced by water rather than originating from the soil seed bank.

In addition, these areas contain a high level of soil nutrients due to the continuous deposition of sediment during the flooding period [40]. In case of an abandoned field, because the soil nutrients were almost accumulated in the litter layer on the surface of ground in the early stage of succession, the nutrient flow from litter to soil by decomposition of litter helps the introduction and development of late-successional plant species [37]. This means the soil nutrient content has great effect on vegetation development by each stage of succession. Thus, it is possible that the process of primary succession in the exposed area occurred at a faster rate than what would be expected for secondary succession in a terrestrial area with relatively low soil fertility.

Given that the cause of gravity-dispersed plant introduction was running water, the distribution of exotic plants in the exposed floodplains in this study was attributed to dispersal by both water and animals (Figure 3 and Figure 4). However, animals contributed more to the dispersion of exotic plants than native plants. Similarly, the previous report on an abandoned paddy field, in the early stage of secondary succession, revealed that 86% of the dispersal of exotic plants in that region was due to water, while 14% was due to animals and 94% of the dispersal of native plants was due to water, while 6% was due to animals [37]. These results demonstrate that animals play a role in the dispersal of exotic plants.

The early stages of primary succession observed in landscape and vegetation research may be explained by the introduction of exotic plants by running water. However, the number of gravity-dispersed plant species did not correlate with the number of mechanically dispersed plant species (Figure 8). This may be due to the very strong dependence on mechanical dispersion by specialized fruits that accumulate elastic energy during drying [38].

The number of wind- or water-dispersed plant species increased in proportion to the number of runner and rhizomatous plants (Figure 8). This probably occurred due to the potential for these types of plants to be dispersed by running water in the form of propagules, as well as seeds [41]. Dispersal by water helps seeds reach areas of stable germination, relatively simplifying the development process [7], thus substantially contributing to the formation of early pioneer plant communities, including runners and rhizomatous plants in the floodplains [7,41].

Vines displayed a negative correlation with runner plants (Figure 8). They strategically adapt to light through various forms, such as tendril climbers, petiole climbers, stem twiners, and root climbers, while growing vertically and horizontally [42,43]. Similarly, stolons grow horizontally so as to explore environments with abundant resources suitable for growth and vegetative propagation [42]. Therefore, even among the early pioneers, vines and runners can compete for light. However, development of vine stems in *Calystegia sepium* (L.) R. Br. is a function of the amount of soil nutrients present in the total biomass [42]. Thus, in fertile floodplains, soil nutrients and light play a crucial role in the interspecies competition for growth and nutrient acquisition between vines and runners [44]. Therefore, the development of runner plants can indirectly inhibit the development of exotic plants by suppressing the introduction of vine plants.

The number of animal-dispersed plant species increased in proportion to the number of wind-, water-, gravity-, and mechanically dispersed plant species (Figure 8). Therefore, animals can help spread a variety of plants within a newly exposed area. In addition, since the number of animal-dispersed plant species increased in proportion to the number of vine plants, the main cause of vine plant introduction might be animal activity (Figure 8), specifically bird activity. Analysis of the vegetation area of exotic and vine plants, habitat characteristics of birds, and the number of birds according to body size revealed that the wider the exotic vegetation area, the wider the vegetation area of the vine plant and the higher the number of birds (Figure 7).

The vegetation area of the vine plant increased with the decrease in the body size of terrestrial birds (Figure 7). Among small birds, approximately five times more terrestrial birds were observed than waterbirds (Table S3). A study on *Hemiphaga novaeseelandiae* Gmelin showed that the birds migrate farther because of their relatively large body mass [30]. Additionally, the longer the time it took for the seeds to pass through the bird’s stomach, the farther the plant seeds spread [30]. However, the vegetation area of vine plants was not related to the body size of waterbirds, and the lesser the area of exotic plants, the higher the number of large waterbirds (Figure 7). This is probably due to the fact that waterbirds are mainly active in and around water and thus, are more affected by running water and water levels than they are by vine plants. Therefore, even if the seeds of exotic plants were dispersed by waterbirds, their development might be hindered by high water levels.

Small birds were also important in dispersing seeds to nearby floodplains; they achieved this by staying longer around the floodplains. Moreover, their smaller sizes allowed them to navigate easily through the narrow spaces of vine patches. The prominent native small terrestrial bird species, *Passer montanus* (Linnaeus) and *Paradoxornis webbianus* (Gould), are dominant in South Korea [45,46,47,48], accounting for approximately 52% of the total bird population in this study. *P. montanus* and *P. webbianus* are known to flock together and prefer dense, shrubby patches [49,50,51]. An increase in the passerine diversity of the vine patches was reported along the herbaceous cover of the vegetation compared to the bare areas [52]. Thus, the vine patches developed in the floodplains can be deemed suitable for small birds. The results thus show that small bird species in the East Asian floodplains play a critical role in seed dispersal in the early stages of vegetative succession.

In summary, the inflow of small resident terrestrial birds was the main factor that contributed to exotic plant development via the dispersal of vine plants and the expansion of the vine plant vegetation area in the exposed floodplains. On the contrary, runner plants, such as *Phragmites japonica* Steud. and *Echinochloa caudata* Roshev., which are common native plants in South Korea, can be introduced by running water. These plants are relatively unsuitable as landing sites for small birds, and they also compete with vine plants, thereby indirectly suppressing exotic plant dispersion.

Based on these results, it is important to reduce the residence time of small terrestrial birds in order to prevent the inflow of exotic plants as much as possible in newly exposed areas due to a decrease in water level. Because small terrestrial birds can use vines as habitats, efforts to reduce the area of vine patches can be considered as part of a management strategy. It is also necessary to protect areas of runner plant patches that compete with vines. Additionally, in the case of afforestation projects around a riverside, planting trees rather than shrubs, which are often inhabited by small passerine birds [53], can be considered as one of the management strategies.

## 5. Conclusions

This study aimed to identify the causes of the introduction and establishment of exotic plants in newly exposed floodplains formed by the opening of weirs and to suggest a feasible management plan for these areas. Analysis of the landscape, vegetation, flora, including seed dispersal methods, and avifauna revealed the probable causes of exotic vegetation development, and these results were interpreted based on plant type and bird population characteristics. In the early stages of primary succession, exotic plants were mainly flowed into the area by running water (Figure 4). Additionally, they were affected by vine plants, small resident terrestrial birds inhabiting their patches, and runner plants that competed with them in the area exposed by the opening of the weirs (Figure 7 and Figure 8).

Runner plants, a common native plant including *P. japonica* in South Korea, belonging to temperate regions within a river basin [54], adversely affected the exotic plant development indirectly via competition with vines (Figure 8). Therefore, in order to manage exotic plants in this environment, it is necessary to control small resident bird density by maintaining and managing these runner plant patches, removing dense vine patches, such as *Pueraria lobata* (Willd.) Ohwi, *Humulus japonicus* Siebold & Zucc., and *S. angulatus*, minimizing the area of shrub patches, and transplanting common tree- or subtree-layered plants around floodplains.

## Figures and Tables

**Table 1:** The results of DAPC analyses: correlation between five variables of the vines and bird property and principal components (PC).

Items	Abb.	PC 1	PC 2
Ratio of vine area per total vegetation area	Vine	−0.85	−0.35
No. of waterbird individuals	NWB	−0.90	−0.18
No. of terrestrial bird individuals	NLB	−0.82	0.35
Body size of waterbird species	SWB	0.35	−0.86
Body size of terrestrial bird species	SLB	0.14	0.93

## Data Availability

The data presented in this study are available in supplementary material.

## Supplementary Materials

The following supporting information can be downloaded at: https://www.mdpi.com/article/10.3390/biology12050696/s1, Table S1: List of dominant species observed around the floodplains of Gongju, Sejong, and Seungchon weirs in autumn 2020; Table S2: Exotic species appearing in seed banks, collected within the high and low levels of floodplains; Table S3: List of avifauna observed around the floodplains of Gongju, Sejong, and Seungchon weirs in 2018.
