# Supplementary material for: Early Exotic Vegetation Development Is Affected by Vine Plants and Bird Activity at Rapidly Exposed Floodplains in South Korea
Source: Biology (Basel). 2023 May 9;12(5):696. doi: 10.3390/biology12050696 (PMC10215534; doi:10.3390/biology12050696)
Supplement: Supplementary file 1 [file biology-12-00696-s001.zip › biology-2361570-supplementary.pdf]

Supplementary data

Table S1. List of dominant species observed around the floodplains of Gongju, Sejong, and Seungchon weirs in Autumn 2020

Vegetation property	Family	Dominant species	Abb.	Life form	Area (m^2)	Ratio (%)
Native vegetation	Cannabaceae	*Humulus japonicus*	Hj	Vine	3,105	0.5
	Leguminosae	*Pueraria lobata*	Pl	Vine	714	0.1
		Lespedeza cuneata	Lc	Perennial	20,472	3.2
	Compositae	*Artemisia princeps*	Ap	Perennial	2,436	0.4
	Cyperaceae	*Cyperus amuricus*	Ca	Annual	3,362	0.5
	Gramineae	*Phragmites communis*	Pc	Perennial	117,397	18.1
		Phalaris arundinacea	Par	Perennial	1,303	0.2
		Setaria viridis	Sv	Annual	7,464	1.1
		*Phragmites japonic*a	Pj	Perennial	18,942	2.9
		Miscanthus sacchariflorus	Ms	Perennial	177,781	27.4
		Pennisetum alopecuroides	Pal	Perennial	12,107	1.9
	Salicaceae	*Salix koreensis*	Sk	Woody	204,207	31.4
		Salix chaenomeloides	Sc	Woody	77,992	12.0
	Moraceae	*Morus alba*	Ma	Woody	818	0.1
	Polygonaceae	*Persicaria nodosa*	Pn	Annual	1,486	0.2
Sub total	-	-	-	-	649,586	17.5
Exotic vegetation	Cucurbitaceae	*Sicyos angulatus*	Sa	Vine	11,892	16.2
	Leguminosae	*Vicia villosa*	Vv	Vine	1,467	2.0
		Robinia pseudoacacia	Rp	Woody	23,392	31.9
	Hamamelidaceae	*Liquidambar styraciflua*	Ls	Woody	916	1.3
	Compositae	*Xanthium strumarium*	Xs	Annual	1,687	2.3
		Coreopsis lanceolata	Cl	Perennial	33,967	46.3
Sub total	-	-	-	-	73,321	2.0
Plantation	-	Shrub	-	-	44,472	12.1
	-	Tree and sub tree	-	-	322,829	87.9
Sub total					367,301	9.9
Sand bar	-	-	-	-	273,122	7.4
Artificial structure	-	-	-	-	646,936	17.5
Water body	-	-	-	-	1,691,195	45.7
Total	-	-	-	-	3,701,461	100.0

Table S2. Exotic species appearing in seed banks, collected within the high and low levels of floodplains

Family	Scientific name	No. of ind. In the low level	No. of ind. In the high level
Amaranthaceae	*Celosia cristata* L.	-	1
Brassicaceae	*Lepidium apetalum* Willd.	-	7
	Thlaspi arvense L.	-	1
Chenopodiaceae	*Chenopodium ficifolium* Smith	24	27
Compositae	*Erigeron annuus* Pers.	18	31
	Senecio vulgaris L.	1	-
	Ambrosia trifida L.	1	77
	Bidens frondosa L.	3	2
	Lactuca scariola L.	-	41
Convolvulaceae	*Quamoclit coccinea* Moench	-	1
Gramineae	*Bromus tectorum* L.	2	-
	Panicum dichotomiflorum Michx.	12	-
Leguminosae	*Trifolium repens* L.	20	-
	Phaseolus vulgaris var. *humilis* Alef.	-	1
Onagraceae	*Oenothera biennis* L.	-	2
Phytolaccaceae	*Phytolacca americana* L.	-	1
Polygonaceae	*Rumex crispus* L.	232	85
Rosaceae	*Potentilla supina* L.	3	16
Scrophulariaceae	*Veronica arvensis* L.	4	25

Table S3. List of avifauna observed around the floodplains of Gongju, Sejong, and Seungchon weirs in 2018

Family	Scientific name	Korean name	Status[a,b]	No. of ind.	Body size[b] (cm)	Sorting by size[c]
Anatidae	*Aix galericulata* (Linnaeus)	원앙	Res	64	45	Large
	Anas poecilorhyncha Forster	흰뺨검둥오리	Res/WV	178	61	Large
Ardeidae	*Butorides striata* (Linnaeus)	검은댕기해오라기	SV	1	52	Large
	Egretta garzetta (Linnaeus)	쇠백로	SV	77	61	Large
	Ardea cinerea Linnaeus	왜가리	Res	112	93	Large
	Ardea alba Linnaeus	중대백로	SV/WV	86	90	Large
	Egretta intermedia (Wagler)	중백로	SV	2	68	Large
	Nycticorax nycticorax (Linnaeus)	해오라기	SV	2	57	Large
	Bubulcus ibis (Linnaeus)	황로	SV	62	50	Large
Charadriidae	*Charadrius dubius* Scopoli	꼬마물떼새	SV	2	16	Small
Columbidae	*Streptopelia orientalis* (Latham)	멧비둘기	Res	45	33	Small
	Columba livia domestica	집비둘기	Res	178	32.5	Small
Coraciidae	*Eurystomus orientalis* (Linnaeus)	파랑새	SV	8	29.5	Small
Corvidae	*Pica pica* (Linnaeus)	까치	Res	108	46	Large
	Cyanopica cyanus (Pallas)	물까치	Res	20	37	Small
	Corvus macrorhynchos Wagler	큰부리까마귀	Res	22	57	Large
Cuculidae	*Cuculus canorus* Linnaeus	뻐꾸기	SV	1	35	Small
Emberizidae	*Emberiza cioides* Brandt	멧새	Res	1	16	Small
Falconidae	*Falco subbuteo* Linnaeus	새호리기	SV	1	34.25	Small
	Falco tinnunculus Linnaeus	황조롱이	Res	7	35.75	Small
Hirundinidae	*Cecropis daurica* (Laxmann)	귀제비	SV	2	19	Small
	Hirundo rustica Linnaeus	제비	SV	27	18	Small
Laniidae	*Lanius bucephalus* Temminck & Schlegel	때까치	Res	3	20	Small
Motacillidae	*Motacilla grandis* Sharpe	검은등할미새	Res	2	21	Small
	Motacilla alba Linnaeus	알락할미새	Res	2	19.5	Small
Muscicapidae	*Phoenicurus auroreus* (Pallas)	딱새	Res	2	14	Small
Oriolidae	*Oriolus chinensis* Linnaeus	꾀꼬리	SV	4	26	Small
Paridae	*Parus major* Linnaeus	박새	Res	22	14	Small
Passeridae	*Passer montanus* (Linnaeus)	참새	Res	441	14.5	Small
Phalacrocoracidae	*Phalacrocorax carbo* (Linnaeus)	민물가마우지	Res/WV	21	82	Large
Phasianidae	*Phasianus colchicus* Linnaeus	꿩	Res	2	70	Large
Picidae	*Dendrocopos kizuki* (Temminck)	쇠딱다구리	Res	2	15	Small
Pycnonotidae	*Microscelis amaurotis* (Temminck)	직박구리	Res	34	34	Small
Scolopacidae	*Actitis hypoleucos* (Linnaeus)	깝작도요	PM	5	20	Small
	Tringa ochropus Linnaeus	삑삑도요	PM	1	23	Small
	Tringa glareola Linnaeus	알락도요	PM	1	20	Small
Timaliidae	*Paradoxornis webbianus* (Gould)	붉은머리오목눈이	Res	292	13	Small
Upupidae	*Upupa epops* Linnaeus	후투티	SV	1	28	Small

a : Res - Resident, SV - Summer Visitor, WV - Winter Visitor, PM - Passage Migrant
b : Lee, W.S.; Koo, T.H.; Park, J.Y.; Taniguchi, T. A field guide to the birds of Korea; LG Evergreen Foundation: Seoul, South Korea, 2014
c : Small - body size ≤ 40 cm, Large - body size > 40 cm
